# Circulating CASK is associated with recurrent focal segmental glomerulosclerosis after transplantation

**DOI:** 10.1371/journal.pone.0219353

**Published:** 2019-07-29

**Authors:** Severine Beaudreuil, Xiaomeng Zhang, Florence Herr, Francis Harper, Jean Jacques Candelier, Ye Fan, Hilal Yeter, Caroline Dudreuilh, Lola Lecru, Aime Vazquez, Bernard Charpentier, Hans K. Lorenzo, Antoine Durrbach

**Affiliations:** 1 IFRNT, Department of Nephrology, Bicêtre Hospital, University of Paris-Sud, Le Kremlin-Bicêtre, France; 2 INSERM U1197, Villejuif, France; 3 CNRS, UMR 8122, Institut Gustave Roussy, Villejuif, France; UCL Institute of Child Health, UNITED KINGDOM

## Abstract

**Introduction:**

Focal and Segmental GlomeruloSclerosis (FSGS) can cause nephrotic syndrome with a risk of progression to end-stage renal disease. The idiopathic form has a high rate of recurrence after transplantation, suggesting the presence of a systemic circulating factor that causes glomerular permeability and can be removed by plasmapheresis or protein-A immunoadsorption.

**Results:**

To identify this circulating factor, the eluate proteins bound on therapeutic immunoadsorption with protein-A columns were analyzed by comparative electrophoresis and mass spectrometry. A soluble form of calcium/calmodulin-dependent serine protein kinase (CASK) was identified. CASK was immunoprecipitated only in the sera of patients with recurrent FSGS after transplantation and not in control patients. Recombinant-CASK (rCASK) induced the reorganization of the actin cytoskeleton in immortalized podocytes, a redistribution of synaptopodin, ZO-1,vinculin and ENA. rCASK also induced alterations in the permeability of a monolayer of podocytes and increased the motility of pdodocytes in vitro. The extracellular domain of CD98, a transmembrane receptor expressed on renal epithelial cells, has been found to co-immunoprecipitated with rCASK. The invalidation of CD98 with siRNA avoided the structural changes of rCask treated cells suggesting its involvement in physiopathology of the disease. In mice, recombinant CASK induced proteinuria and foot process effacement in podocytes.

**Conclusion:**

Our results suggest that CASK can induce the recurrence of FSGS after renal transplantation.

## Introduction

Glomeruli play a fundamental role in the physiology of the kidneys by allowing the filtration of small molecules in the urinary space. Alterations in glomerular organization are of clinical importance in renal diseases, as they can lead to proteinuria and the development of chronic kidney disease. Podocytes are critical in maintaining the function of glomerular filtration through two main interrelated elements: *i)* the interdigitating foot processes, which are actin-based structures, and *ii)* the slit diaphragm, which is a set of transmembrane proteins that spread from adjacent interdigitating foot processes to form a zipper-like scaffold. These structures maintain the organization of the foot processes and are therefore critical to the function of the glomerular-filtration barrier. These interactions are strongly compromised in minimal change disease and focal segmental glomerulosclerosis (FSGS), which are associated with structural and functional alteration of podocytes, such as the effacement of foot processes leading to proteinuria in those patients [1].

FSGS accounts for 20% of all cases of nephrotic syndrome in both children and adults [2,3]. FSGS may be secondary to various diseases (e.g., obesity, HIV infection), associated with gene mutations in proteins involved in the organization of foot processes such as podocine, nephrin, CD2AP, alpha actinin-4 [4], or idiopathic. This idiopathic form of FSGS has a poor prognosis and can progress to end-stage renal disease within 3–7 years. This form relapses in 30–50% of cases after a first renal transplantation (rFSGS) and in up to 90% after a second graft [5]. Its occurrence after vaccination and viral infection and its sensitivity to immunosuppressive drugs suggest that the misregulation of the immune system may participate in the development of this disorder [6,7].

The existence of a soluble factor of permeability (SFP) that causes glomerular protein leakage has been proposed because either plasmapheresis or protein-A immunoadsorption rapidly reduces proteinuria [8,9]. In addition, the injection of plasma from patients with rFSGS or eluates from protein-A immunoadsorption induces proteinuria in experimental animals [9–11], suggesting that SFP binds to protein-A columns. Several SFPs, including suPAR, cardiotrophin-like cytokine-1, and autoantibodies such as anti-CD40, have been described, but their validation as causal agents or biomarkers for rFSGS has not been completed [12–15].

In this study, using mass spectrometry (MS), we analyzed proteins eluted from protein-A columns that were taken from patients with early rFSGS after renal transplantation. We identified the presence of a serum form of calcium/calmodulin-dependent serine protein kinase (CASK), suggesting its involvement in rFSGS. CASK is a membrane-associated kinase that mediates many protein-protein interactions in several cell types, including podocytes [16,17]and neurons [18,19]. Previous studies have demonstrated that an extracellular form of CASK is capable to bind CD98 onto CaCO-2 cells[18–21]. CD98 is expressed at the basolateral domain of intestinal cells and its function depends of the molecules associated with. It can interact with heterodimeric aminoacid transportor (HAT-1) playing together a role as aminoacid transporter but also with different β1 or β3 integrins or CD147 modulating cell adhesion or migration [22]. To our knowledge, the present study provides the first evidence of the presence of an extracellular form of CASK in serum that induces structural and functional alterations in podocytes.

## Materials and methods

### Patients

The clinical characteristics, treatments and evolutionof patients with rFSGS and the clinical characteristics of controls are summarized in Tables 1 and 2 (respectively). Patients were treated by protein-A immunoadsorption as described in the subsequent paragraph.

**Table 1:** Clinical characteristics of patients with post transplantation recurrent FSGS.

Pre transplantation period	
Age at the diagnosis of FSGS	44 (30–53)
Gender (Male/Female)	6/2
Race (Caucasian/Afro-American/Asian/Hispanic)	5/2/1/0
Pre transplantation therapies for FSGS	
Steroids	8
Calcineurin Inhibitors	4
Endoxan/ Chloraminophen/Azathioprine	5
Anti CD20 monoclonal antibody	1
Progression to End stage renal Disease (years)	3.7 (2–7)
Duration of Dialysis	3.85 (2–9)
Transplantation	
Age at Transplantation	45 (40–49)
Type of Transplantation (Living vs non Living Donor)	2/6
Induction treatment	8
Basiliximab / Antithymoglobulin	4/4
Maintenance therapy	
Steroids	8
Calcineurin Inhibitors	8
Mycophenolate Mofetil	8
Delayed Graft Function	3
Rejection	3
Post Transplantation recurrence of FSGS	
Time for recurrence of FSGS after Renal transplantation (days)	7.6 (1–20)
Proteinuria at recurrence (g/day)	6.25 (3–10)
Serum creatinine at recurrence (μmol/l)	305 (150–640)
Tacrolimus trough level at recurrence	15.6 (13–18)
Renal biopsies	
Normal renal structure at recurrence	8
FSGS lesions after recurrence	8
Plasma Exchange (PE)/ Immunoadsorption (IAB)	5/3
Number of Plasma Exchange	15.25 (5–37)
Number of Immunoadsorption	9 (3–18)
Reduction of proteinuria (%)	85.7 (71.4–97.5)
Increase of proteinuria after PE/IAB (n)	4
Anti CD20 monoclonal antibody for FSGS recurrence	3
Evolution	
Graft lost at 1 year	3
Last renal function of functioning kidneys	156 (124–193)
Last proteinuria of functioning kidneys	1 (0.2–2.5°
Complication	
Severe infectious disease	4
De novo diabetes	3

**Table 2:** Clinical and biological characteristics of patients in the different groups.

	Transplant patients			Not Transplant patients			Healthy Donors
	Group 1 rFSGS/Tx (*n* = 8)	Group 2 no-FSGS/Tx (*n* = 7)	Group 3 nrFSGS/Tx (*n* = 7)	Group 4 DN (*n* = 6)	Group 5 MN (*n* = 8)	Group 6 MCD (*n* = 4)	Group 7 HD (n = 8)
Age (years)	44 [30–53]	45 [40–49]	37.5 [28–45]	58.7 [47–68]	68 [56–83]	39 [35–49]	22.3 [20–25]
Gender ratio (male/female)	6/2	5/2	1/6	5/1	7/1	3/1	4/4
Serum creatinine (μmol/L)	305 [150–640]	119 [66–178]	126 [99–170]	198.3 [120–300]	110.4 [58–200]	83.5 [80–87]	na
Proteinuria (g/d)	6.25 [3–10]	0	0.10 [0–0.15]	4.25 [3–6]	7.7 [1–17]	5.4 [5–6]	na
Serum albumine level (g/L)	23.9 [18–30]	38.8 [35.2–41]	42 [40–45]	26.5 [17–29.5]	25.1 [20–28]	19 [14–25]	na
Recurrence of FSGS: after RT (days)	7.6 [1–20]	na	na	na	na	na	na
Tacrolimus trough level (ng/L)	15.6 [13–18]	8,2	12.3 [10–14]	na	na	na	na

The project was approved by the local ethics committee « Comité Consultatif de Protection des Personnes participant à une Recherche Biomédicale » (n°4/010). All patients provided their written informed consent. The informatic file developed for the research was approved by the national commission of informatic and liberty. None of the transplant donors were from a vulnerable population and none of them had declared their opposition for organ procurement accordingly to French law (Loi de Bioethique Article L. 1232–1).

Six groups of patients with different nephropathies or healthy donors (group 7) were tested. Their sera were collected and frozen at -80°C.

#### Transplant patients

Group 1: rFSGS after renal transplantation: Eight patients with rFSGS after renal transplantation were included. rFSGS was defined as the appearance of significant proteinuria after renal transplantation. All patients underwent a graft biopsy procedure to verify the absence of renal parenchymal damage and to rule out an acute rejection. Five of these patients received plasmapheresis, whereas the other three patients received immunoadsorption using protein-A. Sera were collected at the time of the recurrence before plasma exchange or immunoadsorption The immunosuppressive regimen for the transplantation is indicated in Table 1.

Group 2: stable renal transplant patients without FSGS: Seven renal-transplant patients without FSGS were included. Their nephropathies were caused by diabetes mellitus (*n* = 2), rheumatoid purpura (*n* = 3), or unknown mechanisms (*n* = 2). They were treated with tacrolimus, mycophenolate mofetil, and steroids. They did not exhibit acute or chronic rejection. Sera were collected 3 months after kidney transplantation.

Group 3, nonrecurrent FSGS without proteinuria: Seven renal-transplant patients with FSGS but without recurrence of the disease in the first three months post-transplantation were included (nrFSGSF/Tx). They were treated with tacrolimus, mycophenolate mofetil, and steroids. Sera were collected at three months after renal transplantation.

#### Not transplanted patients

Group 4: diabetic nephropathy with nephrotic syndrome: Six patients were included with chronic kidney failure and nephrotic syndrome caused by type-II diabetes(biopsy-proven diabetes glomerulonephropathy). Sera were collected at the time of the renal biopsy.

Group 5: membranous nephropathy (MN): Eight patients with idiopathic MN and without a renal transplant were included. All had biopsy-proven MN. Sera were collected at the time of the biopsy.

Group 6: minimal-change disease (MCD): Four patients with MCD were included. All patients but one were cortico-sensible. Sera were collected at the time of the biopsy and before steroid therapy.

#### Healthy controls

Group 7: healthy individuals: Eight healthy individuals were included as control subjects.

In addition to the immunoabsorption, one control patient without proteinuria and nephropathy was treated with immunoadsorption for acquired autoantibody-mediated von Willebrand’s disease to reduce the risk of bleeding before surgery for acute cholecystitis.

### In vivo immunoabsorption on protein-A columns

Three patients affected with rFSGS were treated by serum immunoadsorption using protein-A cartridges according to the following procedure. Immunoadsorption was performed by centrifugation (BT798, Didecco, Mirandola, Italy) to deliver plasma to two pyrogen-free adsorption cartridges (Immuno-adsorba, Excorim, Lund, Sweden) as recommended by the manufacturer [10,23]. The elution of proteins bound to the protein-A cartridges was achieved with 130 mM sodium citrate (pH 2.2), which was collected and equilibrated. Eluates from the protein-A columns during immunoadsorption were collected from these patients and used for MS.

### Mass spectrometry

Proteins eluted from protein-A columns were resolved by sodium dodecyl sulfate polyacrylamide gel electrophoresis (SDS-PAGE). After PAGE, the gel region corresponding to a differential band was excised, crushed and the proteins were reduced, alkylated, and digested with trypsin, as described previously [24].The resulting peptides were recovered and separated by capillary reverse-phase high-performance liquid chromatography (HPLC) coupled to electrospray-ionization and injected into an LCQ Fleet ion-trap MS as recommended by the manufacturer. The data acquired were used in a search of the National Center for Biotechnology Information Non-Redundant (NCBInr) Protein Database using the Turbo SEQUEST tool. The acceptance threshold was a minimum of two peptides per protein, with a cross-correlation score (Xcorr) of >1.9/2.2/3.75 for singly/doubly/triply charged peptides, respectively, and a delta correlation (deltaCn) threshold of 0.1.

### Production of recombinant CASK

Murine and human CASK sequence share 99,892% of identity (925/926). Only the residue 881 (Isoleucine) in humans is not identical, and correspond to valine in mouse. The sequence alignment is indicated below:

Human 841 ALKVLRTAEFAPFVVFIAAPTITPGLNEDESLQRLQKESD**I**LQRTYAHYFDLTIINNEID 900

ALKVLRTAEFAPFVVFIAAPTITPGLNEDESLQRLQKESD**+**LQRTYAHYFDLTIINNEID

Mouse 841 ALKVLRTAEFAPFVVFIAAPTITPGLNEDESLQRLQKESD**V**LQRTYAHYFDLTIINNEID 900

The cDNA sequence coding for human CASK was kindly provided by Prof. Zenta Walther (Yale University, School of Medicine). DNA was digested with *BamHI* and *EcoRI* and subcloned into a pTrcHis2C vector (Invitrogen) for subsequent expression and purification from *Escherichia coli*. Expression and purification were performed by GenScript services (Piscataway, NJ, USA). The purity was close to homogeneity (>95%), and endotoxins were removed (<0.10 EU/ml). The final protein was dialyzed against phosphate-buffered saline (PBS).

### Serum immunoprecipitation of CASK

Sera were incubated with 25μl of specific anti-CASK (Santa Cruz, H-107, 200 μg/ml) antibody, and were incubated at 4°C overnight in a rotating mixer. Then, protein G-agarose beads were added and incubated at 4°C for 2 h. After centrifugation the supernatant was removed, the beads were washed and the complexes were recovered in Laemmli buffer.

### Cell culture

A conditionally immortalized mouse podocyte cell line was cultured as described by Mundel et al. (21).

### Immunoblotting

Equal amounts of protein (25–50 μg), determined by bicinchoninic acid (BCA assay, Pierce), were separated by SDS-PAGE, transferred to a PVDF membrane, blocked (4% BSA in TBS-Tween) and incubated with primary antibodies for 1 h at room temperature. After three washes, membranes were incubated with secondary antibodies conjugated with horseradish peroxidase, and the activity was detected by enhanced chemiluminescence (Immobilon, Millipore). Each experiment was performed at least three times.

### Immunofluorescence

Conditionally immortalized mouse podocyte cell lines (previously described) [25] were used for the *in vitro* analyses. Podocytes were seeded onto uncoated coverslips Marienfeld GmbH, Germany) and incubated with recombinant CASK (rCASK) (20 μg/ml) for 24 h. After washing in PBS, cells were fixed in paraformaldehyde (3%) for 30 min, washed with PBS, and incubated for 15 min in 100 mM NH_4_Cl in PBS. Cells were permeabilized with 0.05% saponin, incubated for 1 h with primary antibodies (Rabbit Anti-ZO-1 from Zymed Laboratories Inc. Laboratories, South San Francisco, CA) (1: 100 dilution); mouse anti- Synaptopodin (D-9) from Santacruz (sc-515842; 1:50 dilution); mouse anti-mENA from Santacruz (sc-135988, 1:50 dilution); goat anti-Nephrin (N-20) from Santacruz (sc-19000: 1:15 dilution) and mouse anti-Vinculin from Sigma (SAB4200729, 1:100 dilution)washed three times, and incubated with antirabbit and antigoat IgG conjugated with Alexa Fluor 594 or 488 (Invitrogen). Cells were mounted in Mowiol medium, and fluorescence was observed with a Leica DM-RXA23D microscope (Wetzlar, Germany). For tissue staining, frozen kidney sections (4μm) from both groups of mice were processed as described for the cultured cells excepted that no permeabilization was performed.

### Crosslinking processes

rCASK (1 mg/ml) (or serum from patients) was added to the podocytes. After incubation (1 h on ice) and washing, crosslinking was achieved by adding DSS according to the manufacturer's protocols (Thermo Scientific Pierce). After extensive washing, the cells were lysed with RIPA buffer for subsequent immunoprecipitation (as described above).

### Gene silencing

For the CD98 knockdown experiments, we used a pool of three target-specific siRNA oligonucleotides designed to inhibit the expression of CD98. siRNA transfection was performed according to the manufacturer's protocols (Santa Cruz). *CD98* gene expression was abrogated with 100 pmol of siRNA oligonucleotides and the expression of CD98 was evaluated 48 h post-transfection.

### Cell tracking

Podocytes were seeded (500 cells/well) into standard 24-well plates. To explore the effects of CASK, cells were incubated with complete medium (see above) plus 20 μg/ml of recombinant protein. As controls, cells were incubated in the same medium with the same volume of CASK vehicle (PBS). Wells were imaged every 20 min over a 24-h time period using a Zeiss Axiovert 200M microscope, Zeiss AxioCam Mrm camera (Zeiss) and CO_2_-controlled chamber. Each movie was viewed, and individual cells were tracked using Axiovision software. Tracking was performed by marking cell centroids for each 20-min time interval. The collected X and Y centroid coordinates were saved as a data file and input into the ImageJ program to plot displacement and cell motility.

### Podocyte monolayer permeability

Podocytes were plated at a cell density of 100 000/cm^2^ on Transwell polycarbonate membranes (1-μm pore size, 23.1-mm diameter) in triplets for permeability assays. Cells were cultured for 2 days in complete medium until a monolayer was achieved. Then, the nonadherent cells were removed, and the medium was replaced. The effect of CASK was explored by incubation with 20 μg/ml of protein plus 250 μg/ml of mouse albumin. Permeability changes were estimated by analyzing the diffusion of mouse albumin between the upper and lower chambers by collecting samples from the lower chamber at different times. The concentration of mouse albumin was calculated by ELISA (Bethyl Labs). As a control, we added 250 μg/ml of mouse albumin in the absence of CASK.

### In vivo experiments

Animal experiments have been approved by the ethics committee “Consortium des Animaleries Paris SUD” (CAPSUD/N°26). All animal procedures were in accordance with European Union Guideline for the Care and Use of Laboratory Animals. Mice have been subjected to general deep anesthesia with isoflurane by inhalation and then sacrificed by exsanguination and cervical dislocation.

rCASK was produced with a purity close to homogeneity and with no endotoxins. Male wild-type FVB mice (Charles River Laboratories), aged 8 weeks, were administered a single intravenous injection of rCASK (5 μg/g of mouse body weight, *n* = 10) or a similar amount of mouse albumin (controls, *n* = 10). Mice were placed in metabolic cages, and urine was collected for 24 h after the injection. Albuminuria was determined by ELISA (Bethyl Labs). At 24 h after urine collection, the mice were sacrificed, and their kidneys were processed for immunohistochemistry or electron microscopy. The difference between the mean of proteinuria was analyzed by Student’s t test. The glomerular structure has been analyzed by electron-microscopy and the size of foot processes (FP) and the size of slide diaphragm (SD) have been determined sections from 5 different animals per group. The size of the FP was determined by measuring the length of each foot along the glomerular basal membrane. Similarly, SD correspond to the distance between 2 podocyte feet along the glomerular basal membrane. The mean values of FP and SD have been compared between the 2 groups of animals.

## Results

### Identification of CASK in the sera and eluates of patients affected by rFSGS

**Fig 1:**
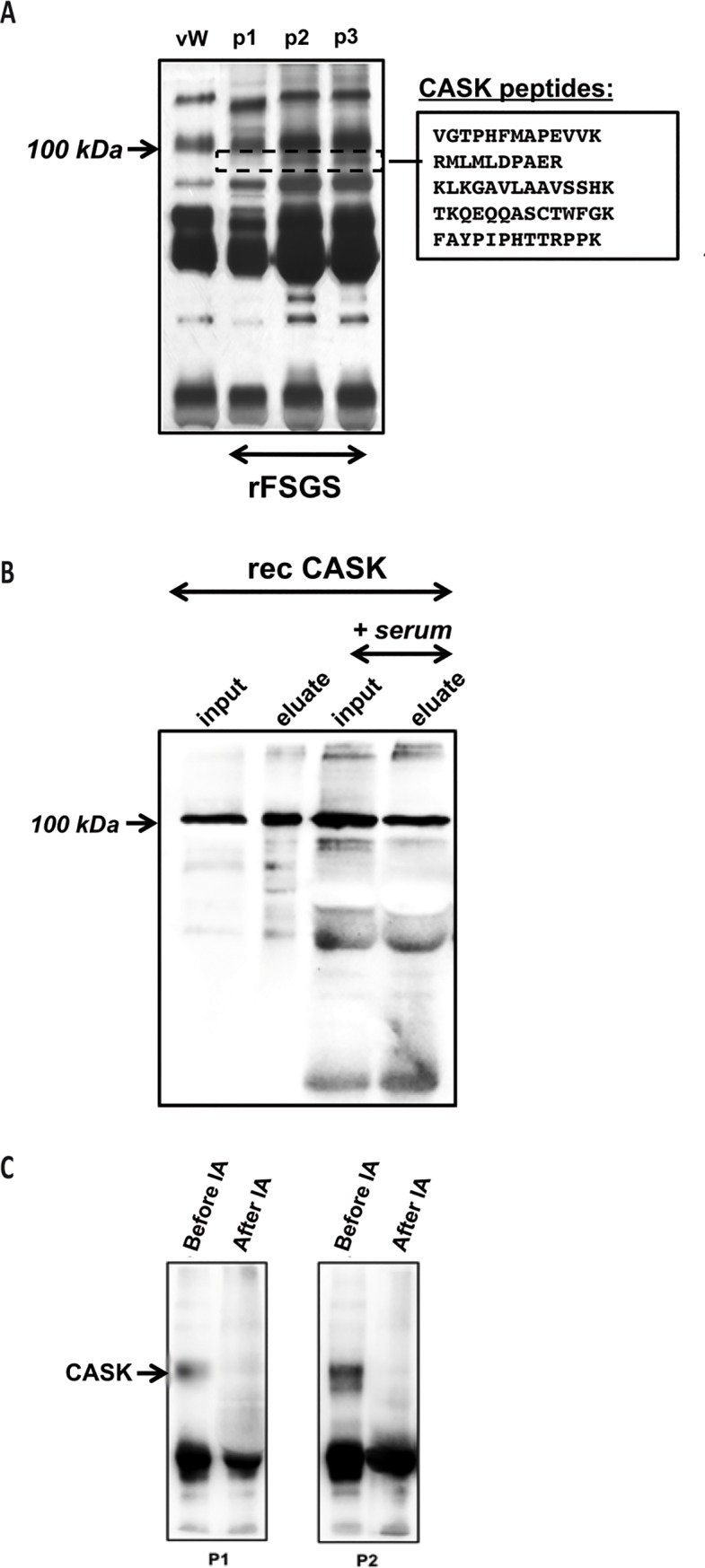
Identification of CASK in the sera of patients affected by rFSGS. A) PAGE of samples from patients with rFSGS or a von Willebrand’s disease and eluted from protein-A columns after immunoadsorption. *Dashed box*: Peptidic sequences identified by MS from three rFSGS patients (p1, p2, p3). *vW*: Patient treated for von Willebrand's disease; P1–P3: three patients having a rFSGS. B) Affinity of CASK to protein-A beads. rCASK binds to protein-A even in the presence of nonspecific human immunoglobulins (in normal serum). All incubated CASK (input) was recovered after the acidic elution of protein-A (eluted). The presence of immunoglobulins did not seem to affect this interaction. C) CASK was efficiently removed by protein-A immunoadsorption from sera from rFSGS patients. CASK was only observed in rFSGS sera before immunoadsorption and was depleted after the procedure. CASK was evaluated by anti-CASK Western blot analysis.

Three patients with rFSGS after renal transplantation were treated by serum immunoadsorption using protein-A columns as soon as the diagnosis of recurrence was made. Immunoadsorption led to significant diminution of proteinuria from 9.45±2.75g/day to 1.19±0.54g/day after 3 sessions of immunoadsorption on protein-A columns (p<0.01). The eluted product from protein-A columns was separated and analyzed by SDS-PAGE and compared with a protein-A eluate from one patient treated for von Willebrand’s disease and with no proteinuria (control). All rFSGS patients displayed a differential band with an apparent molecular weight of ~100kDa (Fig 1A). The band was processed by MS for identification. Five different peptides (see Fig 1A), which complied with the acceptance threshold (see Materials and Methods), were identified and corresponded to CASK a calcium/calmodulin-dependent-serine protein kinase which participates to the cytoskeleton scaffold in many cells and have been demonstrated to bind to the extracellular domain of CD98.

We confirmed that CASK was able to bind protein-A by incubating rCASK in the absence or presence of protein-A-Sepharose beads (Fig 1B). In addition, CASK was only observed in rFSGS sera *before* protein-A immunoadsorption and it was efficiently removed by incubating sera with prot-A conjugated with sepharose beads (Fig 1C).

**Fig 2:**
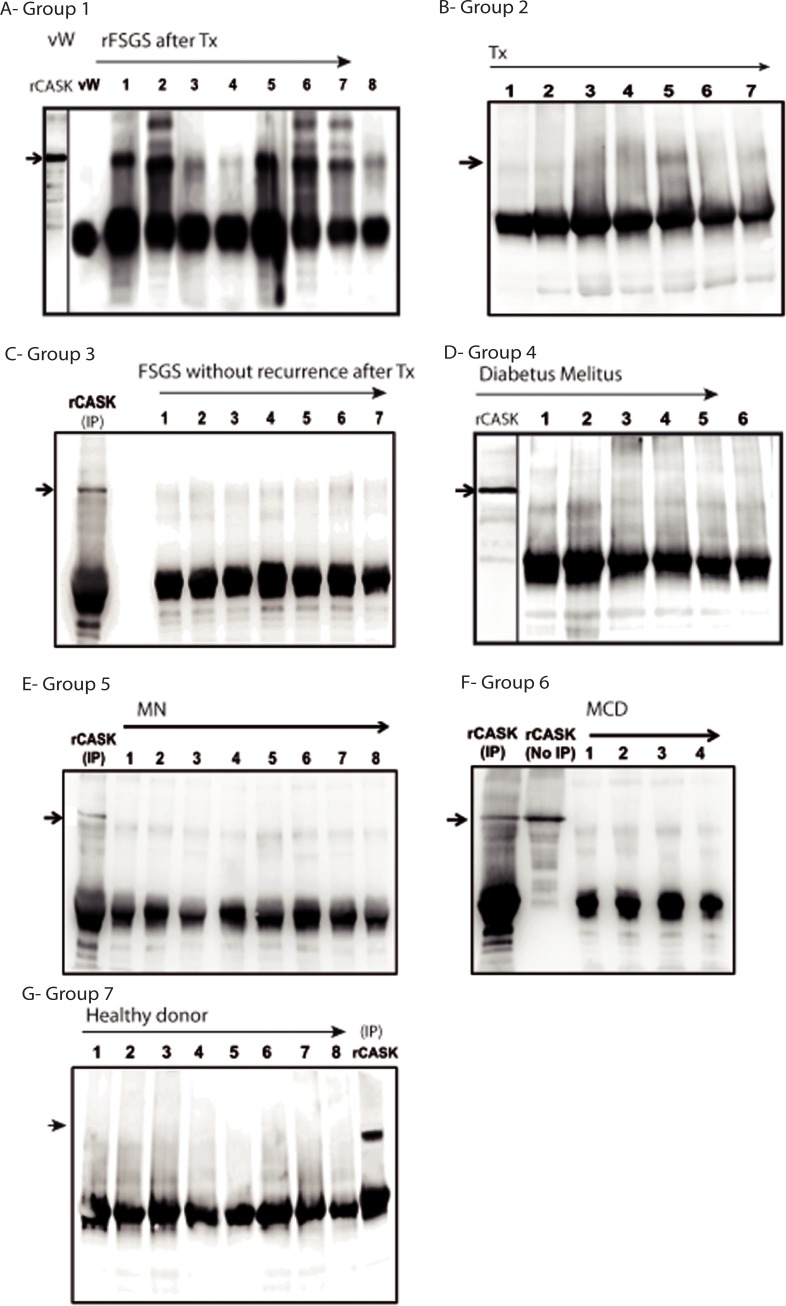
CASK immunoprecipitation in patient sera. A) CASK was only identified in rFSGS patients. wW (A) corresponds to the serum from a patient with an anti-von Willebrand antibody and secondary antibody treated by immunoadsorption. B) renal-transplant patients without proteinuria (Trx), or C) kidney-transplant FSGS patients without recurrence of FSGS after transplantation. In addition, CASK was not detected in the sera of patients with significant proteinuria (>3 g/day) caused by D) diabetic nephropathy, E) membranous nephropathy (MN), F) minimal-change disease (MCD). G) CASK was not detected in healthy donors (positive control: 10 ng of recombinant human CASK (rCASK) diluted in the serum of the healthy individual [n° 5]).

Eight patients with rFSGSoccuring rapidly, during the first three weeks after renal transplantation have been studied. They were treated by immunoadsorptions or plasma exchanges resulting in a consistent strong reduction of proteinuria for all of them (median proteinuria reduction 85%) (Table 1). Threepatients had a relapse of proteinuria when immunoadsorptions or plasma exchanges were stopped because of severe infection and they lost their graft several weeks after. We tested for the presence of CASK in sera from rFSGS patients (n = 8) by immunoprecipitation (Fig 2A). A band was detected for all these patientsWe tested the presence of CASK in 6 other groups of patients. Demographic data are summarized in Table 2. CASK was not detectable in the control (von Willebrand’s disease; Fig 2A), in non-FSGS-related renal-transplant patients (Fig 2B), or renal-transplant FSGS patients who did not exhibit recurrence after transplantation (Fig 2C). We did not detect CASK in the sera from patients with significant proteinuria (>3 g/day) in diabetic nephropathy (Fig 2D) or caused by membranous nephropathy (Fig 2E), and minimal change disease (Fig 2F), or in healthy individuals (Fig 2G).

### Effects of rCASK on the membrane and cytoskeleton of podocytes

**Fig 3:**
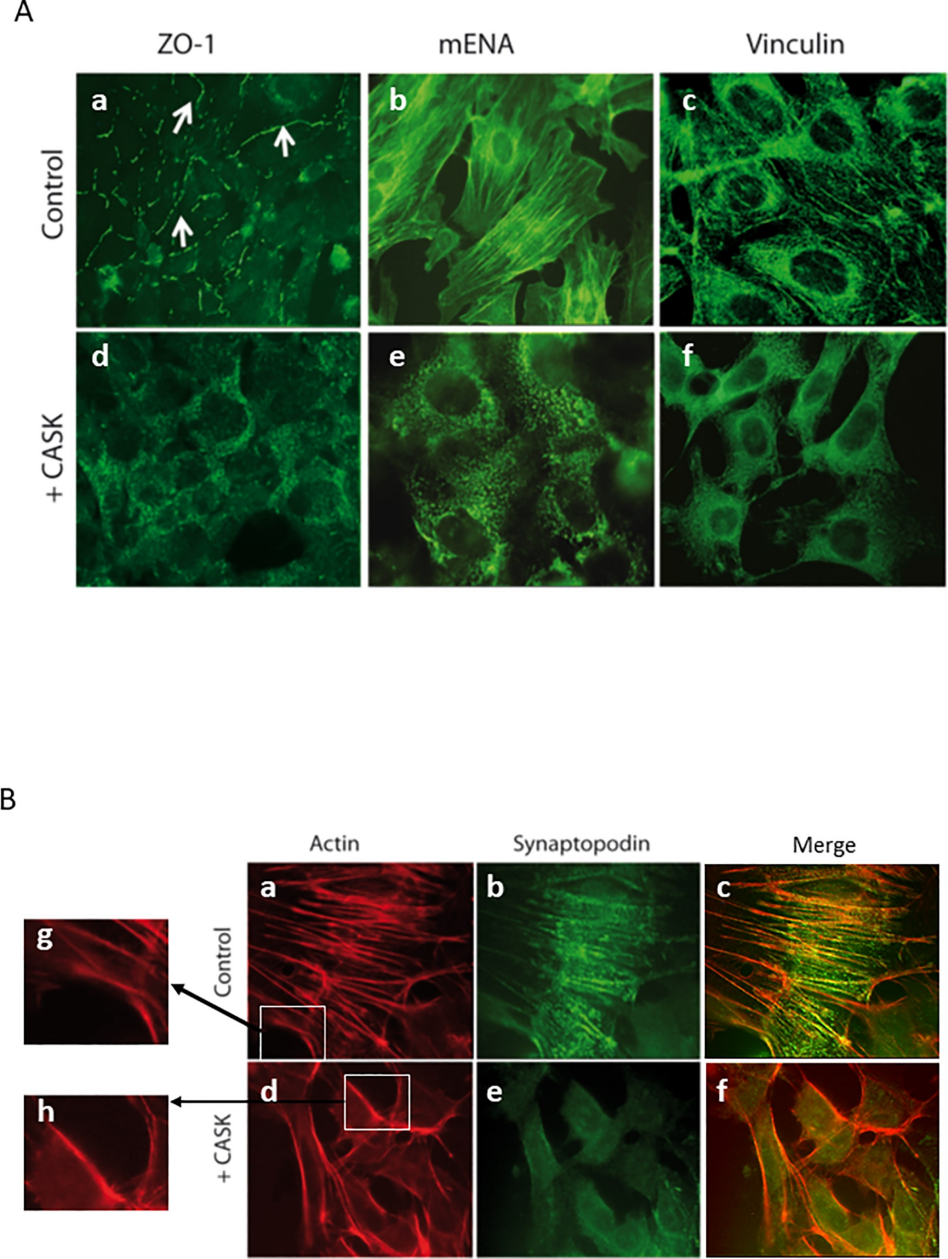
CASK induced structural alterations in cultured podocytes. A) Recombinant human CASK (10 μg/ml) (d,e,f) added to the medium culture affects the distribution of ZO-1, which lost its membrane localization (*arrows*) and developed a cytosolic distribution following a punctate profile (a,d). rCASK also affects the distribution of mENA (b,e) and vinculin (c,f), which are proteins associated with actin structures. B) Recombinant human CASK (rCASK) added to the culture medium (d,e,f,h), impairs the podocyte cytoskeleton (a,c,d,f,g,h). The fibrillar actin pattern was lost after 24 h of incubation with rCASK, but F-actin underneath the plasma membrane remained. Similarly, synaptopodin (b,c,e,f) exhibited a more diffuse pattern after rCASK treatment.

We explored the effect of CASK on the expression and distribution of several membrane proteins in cultured podocytes by adding rCASK to the culture media. CASK had no apparent effect on the expression or distribution of nephrin, p-cadherin, podocin (S1 and S2 Figs) but did affect the cellular localization of ZO-1 (Fig 3A). In the absence of rCASK, the tight junction protein ZO-1 is concentrated along the intercellular membrane in podocytes (Fig 3A). This pattern was disrupted after treatment with rCASK. ZO-1 exhibited a punctate cytoplasmic distribution after 24 h of treatment (Fig 3A).

In addition, the actin cytoskeleton appeared to be significantly affected by rCASK (Fig 3B). In treated cells, the presence of F-actin fibers was reduced, but actin still exhibited a cortical distribution underneath the plasma membrane. Similarly, synaptopodin, an essential protein for the integrity of the podocyte cytoskeleton, mENA and vinculin displayed a linear distribution corresponding to fibrillar staining in control cells; however, after treatment, a more diffuse staining was observed, suggesting the loss of co-association with actin stress fibers in treated cells (Fig 3A).

### Functional impact of CASK in cultured podocytes

**Fig 4:**
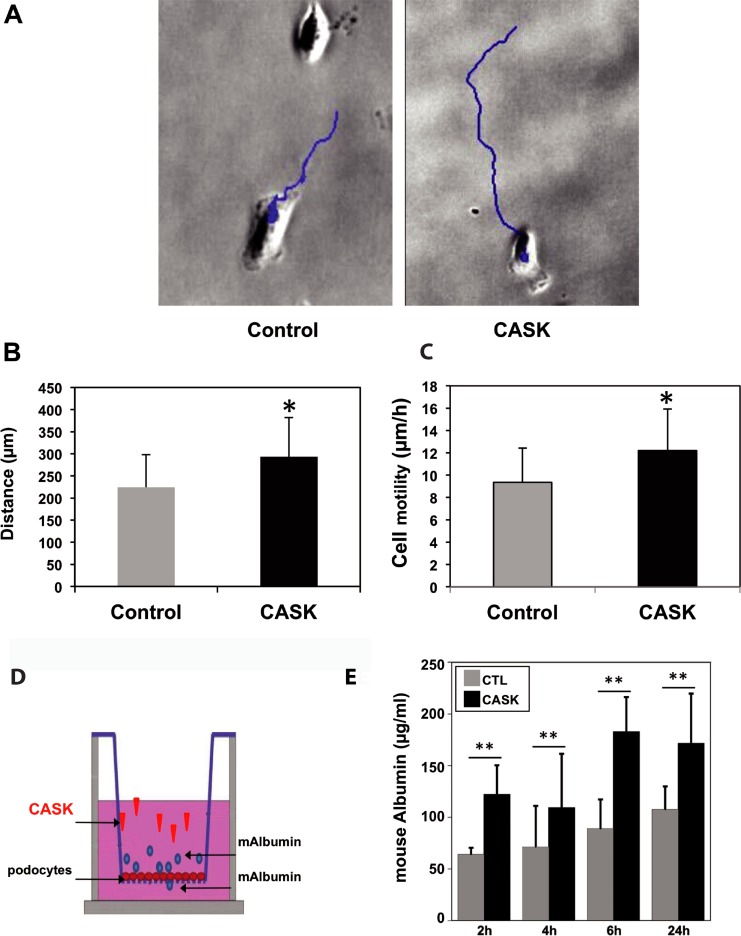
Dynamic alterations induced by CASK in podocytes. A) CASK increased the distance (*blue lines*) and motility of podocytes. B) Distance and C) cell motility were calculated throughout 24 h of incubation with 10 μg/ml of rCASK (*p<0.05, n = 100). D) Schematic of the assay of passive diffusion of mouse albumin through a podocyte monolayer in a Transwell system. Control (PBS-BSA) or rCASK was added to the upper chamber in the presence of mouse albumin, which was measured in the lower chamber by an appropriate ELISA test. E) Compared with the control (*gray bars*), CASK (*black bars*) increased the permeability of the podocyte monolayer after 2 h of incubation; the maximum value was reached at 6 h (*p*<0.05).

Based on the structural modifications induced by CASK in podocytes, we explored the impact of their motility by video-microscopy. After rCASK treatment, isolated podocytes had an increase in motility (12.2±3.7 μm/h) compared to the untreated cells (9.3±3 μm/h) (*p*<0.05, n = 100) (Fig 4A–4C). We tested the integrity of the podocyte monolayers grown on filter supports *in vitro* following treatment with or without rCASK (Fig 4D). Fig 4E shows the ability of podocytes to retain mouse albumin in the Transwell chamber. In the control cells, the concentration of mouse albumin that diffused across the monolayer was only slightly changed over time (up to 100 μg/mL after 24 h) (Fig 4E). In contrast, in rCASK-treated cells, the permeability of the monolayer rapidly increased from 2 h onward and was maintained throughout the experiment (*p*<0.001) (Fig 4E).

### Extracellular CASK-CD98 interactions in podocytes

**Fig 5:**
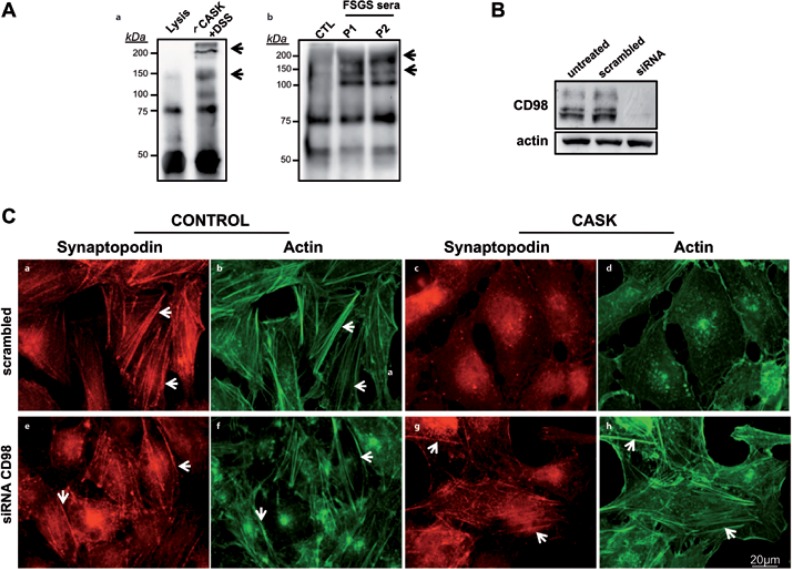
Interaction of CASK with CD98 on the surface of podocytes. A) Coimmunoprecipitation of CASK and CD98 on the membranes of podocytes. Cells were incubated with either recombinant myc-tagged CASK (a) or human sera (b) (one healthy control and two rFSGS patients, see Materials and Methods). Immunoprecipitation with anti-myc (for rCASK, 8% PAGE) or anti-CASK (sera, 10% PAGE), followed by Western blotting with anti-CD98, allowed us to identify a single ~75-kDa band that corresponded to CD98. After crosslinking using DSS, other heavier proteins appeared as different bands, suggesting interactions between CASK and CD98. B) Knock-down of CD98 in podocytes by siRNA. Transfection of a set of siRNA oligonucleotides abrogated the expression of CD98. C) siRNA inhibition of CD98 (e,f,g,h) avoided the alterations (c,d) induced by CASK in podocytes. Fibrillar distribution of actin and synaptopodin (*white arrows*) was maintained after the abrogation of CD98 (g,h) by siRNA even in the presence of CASK.

Previous studies have demonstrated that an extracellular form of CASK is capable of binding CD98 on Caco-2 cells [20,21]). To investigate such an interaction in podocytes, we incubated these cells with either a c-myc-tagged rCASK (Fig 5Aa) or plasma from rFSGS patients (Fig 5Ab). Chemical crosslinking was then performed, followed by immuno-precipitation with anti-myc or anti-CASK antibodies, respectively. After immunoblotting with anti-CD98, we found that, in both cases, CD98 was associated with CASK (Fig 5A). To confirm these results, we knocked down the expression of CD98 by siRNA (Fig 5B). As expected, podocytes transfected with scrambled siRNA (control) displayed an altered cytoskeleton distribution (Fig 5Cd) when incubated with rCASK. In contrast, the actin cytoskeleton was unaffected in podocytes transfected with CD98 siRNA oligonucleotides, even in the presence of CASK (Fig 5Ch). Taken together, our results suggest that CD98 acts as a CASK partner-receptor, which triggers cytoskeletal reorganization induced by CASK in podocytes.

### Serum CASK-induced proteinuria and the effacement of foot processes in mouse podocytes

**Fig 6:**
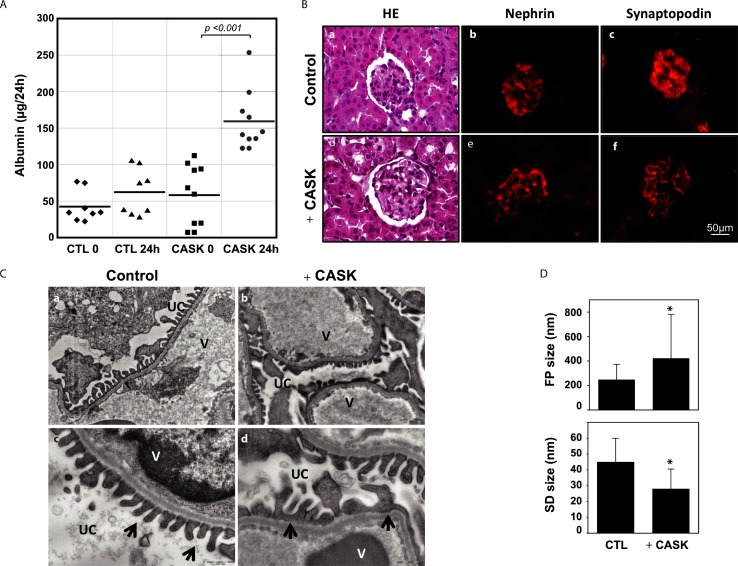
Functional and structural effects of CASK in mice. A) A single injection of CASK induced significant proteinuria compared with levels at time 0 (CASK 0, *p*<0.001) or those of the controls (mouse albumin, *p*<0.0001) after 24 h. B) Microscopy of renal biopsy stained with hematoxylin-eosin of control mice (a) or mice treated with rCASK (d) respectively. Immunofluorescence microscopy analyses of podocytes in control mice or mice treated with rCASK with antibodies against nephrin (b,e) or synaptopodin (c,f). We did not observe any significant alterations in glomerular structures based on hematoxylin-eosin staining or immunofluorescence of cytoskeleton proteins (actin/synaptopodin). C) A single IV injection of rCASK induced ultrastructural modifications in the glomeruli. Electron microscopy revealed effacement of the foot processes, giving the appearance of fusion (*arrows*) (b,d) but not in control (a,c). UC: urinary chamber V: vessel. D) The sizes of the foot processes (FP) and the slit diaphragms (SD) were measured (right images): CASK significantly increased the size of foot processes, while the slit diaphragms were reduced (*p<0.05).

We tested the effects of rCASK in mice. As shown in Fig 6A, no relevant albuminuria was found in the control mice, whereas a significant increase in proteinuria was observed after a single IV injection of CASK (*p*<0.005) (Fig 6A). Histochemistry of the kidneys from the two groups did not reveal any significant differences, namely, no significant deposits, fibrosis, or proliferative lesions were observed (Fig 6B). However, when the kidneys were analyzed by electron microscopy, the CASK-treated mice had clearly developed an altered foot process and had larger foot processes than the control mice, where these structures remained intact (Fig 6C). The size of the foot processes was significantly increased in the mice treated with CASK compared with that in the controls (422.0±30.52nm vs. 248.1±10.03nm, *n* = 145 and 165 measurements, respectively; *p*<0.0001) (Fig 6D). In contrast, the inter-foot process spaces were decreased in mice injected with CASK (28.27±1.959 vs. 43.97±1.160nm, *n* = 61 and *n* = 131 measurements, respectively; p<0.0001), suggesting that CASK induced significant alterations in the foot processes (Fig 6D).

## Discussion

FSGS is the histopathological expression of different variants of nephrotic syndrome. The idiopathic form can relapse very rapidly after transplantation, suggesting the presence of an SFP that can be removed from the blood of patients through its binding to protein-A columns, leading to a reduction of proteinuria [10]. We used this binding capability of protein-A to identify the SFP(s) using an MS approach. Comparing proteins eluted after protein-A immunoadsorption, we identified a serum form of CASK, which was detected only in the sera of patients with rFSGS, but not in healthy individuals, in kidney-transplant patients without proteinuria, patients with significant proteinuria caused by diabetic nephropathy, minimal change disease, or membranous glomerulonephritis.

Human rCASK induced alterations to murine podocytes *in vitro* and *in vivo*; these alterations were associated with proteinuria in mice. Other molecules have been proposed as potential SFPs, but their roles are still under debate [13,14]. Recent studies have shown that the urokinase plasminogen-activator receptor (suPAR) is the soluble factor associated with glomerular permeability and is responsible for primary FSGS and rFSGS [26]. However, the specificity of suPAR as a biomarker for FSGS is controversial, as high levels of serum suPAR have also been found in other pathological conditions, regardless of the existence of proteinuria [27], such as HIV-1 or bacterial infections, malaria, and various types of cancer not necessarily associated with nephrotic syndrome [28,29]. More recently, Delville and colleagues, using a peptide-array system, identified a panel of seven antibodies capable of predicting post-transplant FSGS recurrence with 92% accuracy [15]. Among them, anti-CD40 caused cytoskeleton alterations in podocyte cell culture and *in vivo* when it was coinjected with suPAR. The authors suggested a costimulatory function for both CD40 and suPAR, but larger studies are required to validate this hypothesis.

Protein-A immunoadsorption *in vivo* induced a decrease in proteinuria in patients with rFSGS, suggesting that SFP is efficiently removed by this procedure [10]. Unlike suPAR [23], CASK was efficiently retained by protein-A columns (Fig 1B), suggesting a high affinity of CASK to protein-A. However, it cannot be excluded that alternative complexes of CASK-Ig are formed *in vivo*.

The existence of a serum form of CASK has not been described before. CASK is a multidomain scaffold protein ubiquitously expressed, mainly in neurons and podocytes. It mediates a link between the extracellular matrix and the actin and therefore participates in the organization of the cytoskeleton. Mutations of CASK have beenassociated with intellectual disability, microcephaly and cerebellar hypoplasia [30]. In addition, it has been observed *in vitro* that CASK interacts with the extracellular domain of CD98 in CaCo2 cell lines and on intestinal sections. CD98 is a type 2 transmembrane protein with a PDZ binding domain in its extracellular part and regulates the heteromeric amino acid transporter (HAT) as well as acts as integrin β1 and β3 co-receptor and might regulates adhesion and migration of cells. HAT acts as an exchanger transferring large cationic, neutral proteins and negatively charged amino acids [31–33]. CD98 also regulates adhesion and migration of cells and the signaling pathway downstream integrin such as the FAK and Akt. In human, CD98 has been shown to be associated with cell proliferation and tumor growth [34–37]. This suggests that destabilization of CD98 could favor protein or amino acid release in urine and podocyte detachment.

In our work, one hypothesis for the presence of CASK in sera could be that serum CASK was released from damaged cells. However, no increase in any other intracellular protein from blood cells or podocytes has been observed in blood samples from patients with rFSGS, which suggests that the presence of CASK does not reflect cell lysis or podocyte injury. Immunosuppressive drugs are known to partially control primary FSGS flare-ups as well as the relapsed form after transplantation [6,9]. Therefore, we speculate that CASK could be produced/secreted by an immune cell subset that requires complete characterization.

In our study, a pathological effect was confirmed by a single intravenous injection of rCASK, which increased albuminuria in mice and induced effacement of the podocyte foot processes. These alterations in podocyte shape require the rearrangement of the actin cytoskeleton, a process that leads to FSGS [38,39]. As we have observed, CASK induced morphological rearrangement of podocytes *in vitro*, as evidenced by the following: *i)* alterations in the permeability of the podocyte monolayers and *ii)* cytoskeleton alterations (reorganization of actin, synaptopodin, mENA or junction proteins such as ZO-1). These alterations are similar to others that are widely described for this disease [38,40–44]. In addition, these alterations induced an increase in cell motility that is in accordance with the observed alterations in stress fibers. However, this effect was observed in the presence of a high concentration (10 μg/ml) of rCASK, which may be related to a low affinity effect induced by CASK.

The presence of an extracellular form of CASK has already been reported *in vitro* and on intestinal sections [20]. CASK has been shown to bind to the basolateral membranes of intestinal epithelial cell lines through direct interaction with the extracellular domain of CD98 (heavy chain) [20]. This protein has been found on the surface of many activated cell types and has been implicated in the regulation of cellular differentiation, adhesion, growth, and apoptosis [45]. However, little is known regarding the mechanisms by which CD98 mediates in such functions. However, it can interact with other transmembrane proteins, such as β1 and β3 integrins and CD147. Accordingly, the involvement of CASK/CD98 in the pathogenesis of FSGS is a very attractive hypothesis that needs to be explored.

In conclusion, we propose that the serum form of CASK is a newly discovered factor that needs to be considered in the etiopathology of FSGS. Herein, we describe, for the first time, a potential pathological system that could be used as a new biomarker or therapeutic target for rFSGS. This is based on the existence of soluble CASK that is potentially secreted into the sera of rFSGS patients. This soluble form of CASK could interact with renal podocytes (perhaps through CD98) at the membrane level to trigger cytoskeleton disorganization and then lead to proteinuria. Further studies will be necessary to validate this new model in greater detail.

## Supporting information

S1 Fig A- Distribution of Podocin in podocytes incubated with or without rCASK for 24 hours. B- Expression of Z0-1, Nephrin, P-Cadherine, CD2AP, Podocin, Actine in podocytes incubated for various times with or without rCASK determined by western blot.
(TIF)

S2 Fig Detection of rCASK in sera of mice, 24 hours after a single IV injection. 3 FVB mice have been injected with 5mg/kg of body weight of rCASK (0.7 mg/ml after being dialyzed in NaCl9°/°°) and 3 FVB mice with 5mg/kg of body weight bovine serum albumine(BSA)(0.7 mg/ml after being dialyzed in NaCl9°/°°) as a control group. Because rCASK had a His tag, rCASK was immunoprecipitated with an anti His Tag (Anti-6X His tag antibody; abcam ab9108) and protein G sepharose beads and then revealed after gel electrophoresis and transfer onto nitrocellulose membrane with either the same antibody Anti-6X His tag(abcam ab9108) followed with a donkey anti rabbit secondary antibody conjugated with HRP (Jackson711.035.152).
(EPS)
