# Supplementary figures and images for: Circulating CASK is associated with recurrent focal segmental glomerulosclerosis after transplantation
Source: PLoS One. 2019 Jul 29;14(7):e0219353. doi: 10.1371/journal.pone.0219353 (PMC6663006; doi:10.1371/journal.pone.0219353)

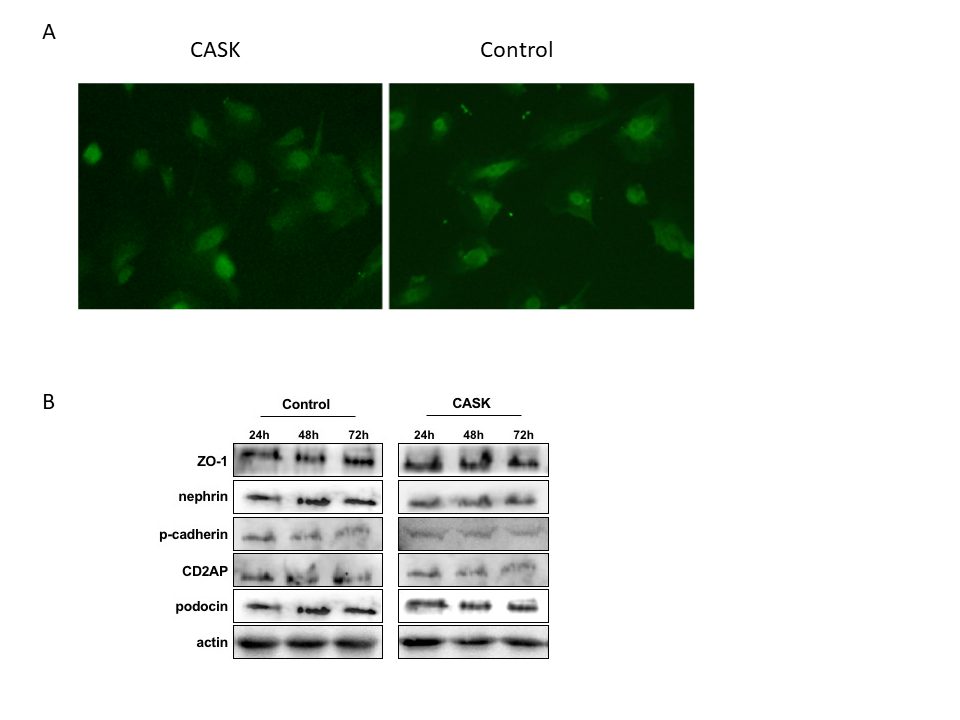

Supplement: S1 Fig — A- Distribution of Podocin in podocytes incubated with or without rCASK for 24 hours. B- Expression of Z0-1, Nephrin, P-Cadherine, CD2AP, Podocin, Actine in podocytes incubated for various times with or without rCASK determined by western blot. (TIF) [file pone.0219353.s001.tif]
